# Plaque burden is associated with minimal intimal coverage following drug-eluting stent implantation in an adult familial hypercholesterolemia swine model

**DOI:** 10.1038/s41598-023-37690-0

**Published:** 2023-07-01

**Authors:** Francesca Razzi, Jouke Dijkstra, Ayla Hoogendoorn, Karen Witberg, Jurgen Ligthart, Dirk J. Duncker, Jan van Esch, Jolanda J. Wentzel, Volkert van Steijn, Gijs van Soest, Evelyn Regar, Heleen M. M. van Beusekom

**Affiliations:** 1 Department of Cardiology, Erasmus MC, Dr. Molewaterplein 40, 3015 GD Rotterdam, The Netherlands; 2 Leiden University Medical Center, Albinusdreef 2, 2333 ZA Leiden, The Netherlands; 3 Department of Chemical Engineering, Delft University of Technology, Van der Maasweg 9, 2629 HZ Delft, The Netherlands; 4 University Hospital Ludwig-Maximilians University, Marchioninistrasse 15, 81377 Munich, Germany; 5 Department of Cardiology, Erasmus MC, University Medical Center, Room Ee2393A, Dr. Molewaterplein 40, 3015 GD Rotterdam, The Netherlands

**Keywords:** Interventional cardiology, Imaging and sensing, Cardiology, Atherosclerosis

## Abstract

Safety and efficacy of coronary drug-eluting stents (DES) are often preclinically tested using healthy or minimally diseased swine. These generally show significant fibrotic neointima at follow-up, while in patients, incomplete healing is often observed. The aim of this study was to investigate neointima responses to DES in swine with significant coronary atherosclerosis. Adult familial hypercholesterolemic swine (n = 6) received a high fat diet to develop atherosclerosis. Serial OCT was performed before, directly after, and 28 days after DES implantation (n = 14 stents). Lumen, stent and plaque area, uncovered struts, neointima thickness and neointima type were analyzed for each frame and averaged per stent. Histology was performed to show differences in coronary atherosclerosis. A range of plaque size and severity was found, from healthy segments to lipid-rich plaques. Accordingly, neointima responses ranged from uncovered struts, to minimal neointima, to fibrotic neointima. Lower plaque burden resulted in a fibrotic neointima at follow-up, reminiscent of minimally diseased swine coronary models. In contrast, higher plaque burden resulted in minimal neointima and more uncovered struts at follow-up, similarly to patients’ responses. The presence of lipid-rich plaques resulted in more uncovered struts, which underscores the importance of advanced disease when performing safety and efficacy testing of DES.

## Introduction

Atherosclerosis, the main cause of coronary artery disease, is the narrowing of coronary arteries due to build-up of fatty deposits in the arterial wall known as plaque^1^. The development of atherosclerosis is the driving cause for arterial interventions using coronary drug-eluting stents (DES), generally being favored over bare metal stents (BMS) to reduce the risk of in-stent restenosis^2^. In coronary BMS, the extent of atherosclerotic plaque prior to stent implantation plays a crucial role in the long-term response of the neointima (NI)^3,4^. In contrast, that role remains unclear and contradictory for coronary DES^5,6^. Healthy or minimally diseased swine coronary models are commonly used for preclinical DES studies^7^, but they usually do not show signs of impaired NI healing as seen in patients, whose coronary arteries are often characterized by uncovered or poorly covered struts at follow-up^8,9^. Instead, healthy models only show fibrotic and homogeneous NI covering stent struts^10,11^. A similar response is seen in atherosclerotic swine models, which, despite their diseased state, generally are relatively young and therefore do not present significant pre-existing plaque burden^12^. Clearly, these models with limited atherosclerosis only allow limited conclusions for patients. We hypothesize that swine models with more extensive pre-existing atherosclerotic plaques will show responses following DES implantation that are more similar to the ones observed in patients, with the extent of atherosclerotic plaque affecting NI responses.

In this study, we used adult familial hypercholesterolemia (FH) swine, in which atherosclerotic plaque develops naturally with the help of an atherogenic diet and without the need for vascular injury^13^. This swine model specifically shows areas with high plaque burden (up to 60%) as well as healthy segments. The chosen adult FH model gives us the opportunity to study NI responses after DES implantation in the presence and absence of relevant plaque and to precisely evaluate how this affects NI responses. Ultrathin Sirolimus-eluting stents were implanted and NI responses were evaluated after 28 days using serial high-resolution intracoronary OCT imaging.

## Materials and methods

### Study design and animals

The animal study protocol was approved by Animals Ethics Committee of the Erasmus University Medical Center Rotterdam, The Netherlands [EMC3125 (109-12-25)]. The study was performed according to the National Institutes of Health Guide for Care and Use of Laboratory Animals and ARRIVE guidelines^14^. We used FH French Bretoncelles-Meishan (FBM) minipigs, homozygous for the LDLR R84C mutation (n = 6, castrated male). They were given a normal laboratory diet (102243/60, Sanders Ouest, Etrelles, France) until the start of the study. At the age of 34 ± 3 months, a high fat diet (10% lard and 0.75% cholesterol, the National Institute of Agronomic Research, France) was given for 9 months to develop atherosclerosis as described before^13^, and the feeding was performed in such a way to keep the animal at a stable weight. Since we expected based on prior work that these adult animals develop plaque with variation in size and composition^13^, ranging from healthy segments to high plaque burden, we have chosen not to include a separate healthy control group. At baseline, blood samples were drawn from the carotid sheath into EDTA and clotting tubes. Standard plasma analysis was performed on fresh plasma by the internal clinical chemical department to determine the levels of total cholesterol. Animals received 300 mg acetyl-salicylic acid and 150 mg clopidogrel 1 day before the intervention and 300 mg acetyl-salicylic acid and 75 mg clopidogrel daily until sacrifice as described before^10^. Animals were sedated i.m. with Xylazine (2.25 mg/kg, 20 mg/mL) and Zoletil 100 (tiletamine/zolazepam; 6 mg/kg, 100 mg/mL), anesthetized with sodium-thiopental (4 mg/kg, 50 mg/mL) i.v., intubated and ventilated with oxygen (25–30% v/v) and nitrogen (75–80% v/v) to maintain blood gases within the physiological range. Anesthesia was maintained by isoflurane inhalation (1–2.5% v/v). Acetylsalicylic acid co-acted as analgesic. During the procedure, 5000 i.u. heparin was administered i.a. per hour. DES (n = 14) were implanted in coronary arteries (n = 14) under guidance of OCT with an intended stent-artery ratio of 1.1:1 at sites of atherosclerotic plaque, as previously described^10,15^. Before imaging, isosorbide mononitrate (0.04 mg/kg, 1 mg/mL) was administered via the guiding catheter to induce epicardial coronary vasodilation. The animals received a thin-strut (64 µm) cobalt-chromium absorbable coating Sirolimus-eluting stent (SES) of 15 mm length (n = 7; MiStent®, Micell Technologies, Inc., Durham, North Carolina) or a thin-strut (60 µm or 80 µm) cobalt-chromium SES of 13 mm (n = 5) or 15 mm (n = 2) length (Orsiro®, Biotronik AG, Bulach, Switzerland). Importantly, both stents show ~ 50% of Sirolimus release at 28 days, to allow investigation of NI responses during stable drug release. Stent selection was not based on pre-existing plaque burden and stents were randomly assigned to the plaques. At 28 days post stent implantation, after the final imaging time point, animals were euthanized by sodium pentobarbital.

### Sequential OCT analysis

OCT pullbacks were obtained pre and post stent implantation, and at 28 days follow-up. The regions of interest were identified at these time points by reviewing pullbacks simultaneously and using anatomical landmarks as references^15^. The analysis of OCT data was performed using QCU-CMS software (version 4.69, Leiden University Medical Centre, LKEB, Division of Image Processing). Each frame (0.2 mm intervals) within the stented segment was serially analyzed. As the importance of studying the edge vascular response has been extensively underlined^16^, the segments 5 mm distal and proximal outside the stent edges were also analyzed.

### Lumen, stent and vessel analysis by OCT

Lumen, stent and vessel [external elastic laminae (EEL)] contours were automatically detected and manually corrected in each frame. The corresponding areas were calculated and averaged per segment of interest for each artery. At post-implant and follow-up, the stent struts, appearing as bright signal-intense structures with dorsal shadowing, were identified placing a marker at their endoluminal side. The apposition of stent struts against the lumen was assessed on a strut-by-strut basis measuring the distance between stent strut and lumen contour, laying on a straight line connecting the marker and the gravitational center of the lumen. Struts were classified as malapposed when the distance between strut marker and lumen contour was greater than the strut thickness itself plus the axial resolution of OCT (14 µm). Struts classified as non-apposed at the ostium of side branches were excluded from strut apposition analysis. At follow-up, struts were classified as uncovered when the thickness of NI coverage was between 0 and 30 µm and hence considered below reliable detection by OCT^17^. For thicker NI, the thickness for each strut was calculated at follow-up. NI burden, calculated as (NI area/stent area)*100%, was measured in each frame and averaged over the stent length. The pattern of coverage was also evaluated, and was classified into homogeneous NI or crenellated NI, as previously described^18^. Homogeneous NI was defined as a smooth NI layer with homogeneous density, while crenellated NI was defined as a corrugated NI layer with non-homogeneous density. Strut coverage type at follow-up was measured in angles for each frame, averaged per stent, and given as a percentage. OCT frames were assessed for signs of neoatherosclerosis.

### Atherosclerotic plaque analysis by OCT

Plaque was evaluated at each frame of the segments (distal 5 mm edge, in-stent, proximal 5 mm edge), allowing precise detection of its contours. Plaque area (difference between the area circumscribed by the EEL and lumen area) was evaluated in each frame at the three different time points, and averaged per segment. In the frames where the EEL was not visible due to lipid presence, we used adjacent frames and the longitudinal reconstruction of the pullback for interpolation. Plaque burden was calculated as (plaque area/EEL area)*100% and evaluated in each frame at the three time points, and averaged per segment. Plaque composition was assessed according to consensus standards^19^ as angles in each frame at the three time points, averaged per segment and given as a percentage. Vessel wall composition was classified as lipid-rich plaque, fibrous plaque, healthy vessel, vessel wall presenting intima irregularities, and other. Angles for these individual tissue components were drawn manually in each frame, averaged per segment and given as a percentage. The definition of the individual tissue components can be found in the Supplementary Methods.

### Atherosclerotic plaque analysis by histology

Coronary samples adjacent to the stented segment were embedded in gelatin, cut into 3 mm blocks, cryosectioned and mounted onto glass slides. In order to allow detection of lipids, the arteries were frozen instead of embedding in a standard matrix. Following confirmation of consistent plaque severity and composition over the wider length of the stented region by OCT, plaques were analyzed 10 mm distal to the stent in order to avoid measuring any influence of stenting on the plaque. Cryosections were stained with Hematoxylin and Eosin (H&E), Resorcin-Fuchsin (RF) and Oil-red-O (ORO) staining to confirm lipid presence and plaque type, showing morphology and composition of a variety of lesions, from healthy segments to lipid-rich plaques. The atherosclerotic plaques were reviewed by an experienced pathologist (HvB) and classified according to the modified plaque classification provided by Virmani et al.^20^: No plaque (NP), Intimal Thickening/Intimal Xanthoma (IT/IX), Pathological Intimal thickening/Fibrous Cap Atheroma (PIT/FCA). Of note, the choice to freeze the arteries instead of embedding it in a standard matrix to allow lipid detection complicates further histological analysis of the stented sections, which is therefore omitted in this study.

### Statistical analysis

Statistical analysis was performed with SPSS (IBM SPSS Statistics 26). Normality was tested with the Shapiro–Wilk test. Groups (ORSIRO and MISTENT) were compared at each time point using a Student’s t-test. *p* < 0.05 was considered significant. Simple linear regression analysis was performed to describe NI burden, percentage uncovered struts and pattern of coverage based on plaque burden, percentage lipid-rich plaque and percentage fibrous plaque.

## Results

Information about swine blood cholesterol levels and weight can be found in Supplementary Table S1.

### Morphometric characteristics

In total, 14 lesions from coronary arteries of 6 swine were stented. Serial OCT imaging was analyzable in all arteries. The EEL was not visible over 360° of the vessel circumference in only 7% (73 of 1074) of analyzed frames. One stent showed a long dissection resulting in extensive malapposition post-implantation (Supplementary Fig. S1). This stent was considered as an outlier and excluded from further analysis. Table 1 shows the OCT in-stent analysis results at pre-implant, post-implant and 28 days follow-up. Data for both stent types are combined in this table, because we found no significant differences between ORSIRO and MISTENT for all the reported parameters (Supplementary Table S2). Given that we did not find differences between ORSIRO and MISTENT, the data for both stents are presented together in this manuscript. Table 2 summarizes the results of the strut-by-strut in-stent analysis in the 13 analyzed stents. At follow-up, in both in-stent and stent edges, no significant differences were found between ORSIRO and MISTENT for the reported parameters (Supplementary Tables S3 and S4). No significant difference was found between the segments (distal edge, in-stent, proximal edge) analyzed for lumen area and plaque burden as measured baseline (post-implantation) and at follow-up (Supplementary Fig. S2).

**Table 1:** Optical coherence tomography (OCT) in-stent analysis of all stent data together pre-implant, post-implant and at 28 days follow-up.

	Variables analyzed	Stent segments
Pre-implant	n	13
	OCT frames	82 ± 28
	Min lumen area (mm^2^)	5.8 ± 1.5
	Mean lumen area (mm^2^)	7.0 ± 1.2
	% plaque burden	40 ± 15
Post-implant	n	13
	OCT frames	126 ± 43
	Min lumen area (mm^2^)	7.8 ± 1.2
	% acute gain	25 ± 13
	Mean lumen area (mm^2^)	8.0 ± 1.4
	Mean stent area (mm^2^)	8.1 ± 1.4
	Min stent area (mm^2^)	6.8 ± 1.3
	% plaque burden	36 ± 13
28 days follow-up	n	13
	OCT frames	98 ± 41
	Min lumen area (mm^2^)	6.5 ± 1.2
	Mean lumen area (mm^2^)	6.6 ± 1.2
	Mean stent area (mm^2^)	7.2 ± 1.2
	Min stent area (mm^2^)	6.6 ± 1.4
	Late loss (%)*	13 ± 18
	% plaque burden	37 ± 10
	% NI burden	9.0 ± 4.0

Results are reported as mean ± standard deviation.

*NI* neointima.

*Negative values were present as some arteries showed a larger lumen area at follow-up as compared to post-implant.

**Table 2:** Strut-by-strut optical coherence tomography (OCT) in-stent analysis of all stent data together pre-implant, post-implant and at 28 days follow-up.

	Variables analyzed	Stent segments
Post-implant	n struts	17,297
	% malapposition	0.8 (0.1–17.7)
28 days follow-up	n struts	13,960
	NI thickness (µm)	110 (72–204)
	% uncovered struts	10.9 (0.6–27.1)

Results are reported as median (min–max).

*NI* neointima.

### Plaque histology

**Figure 1:**
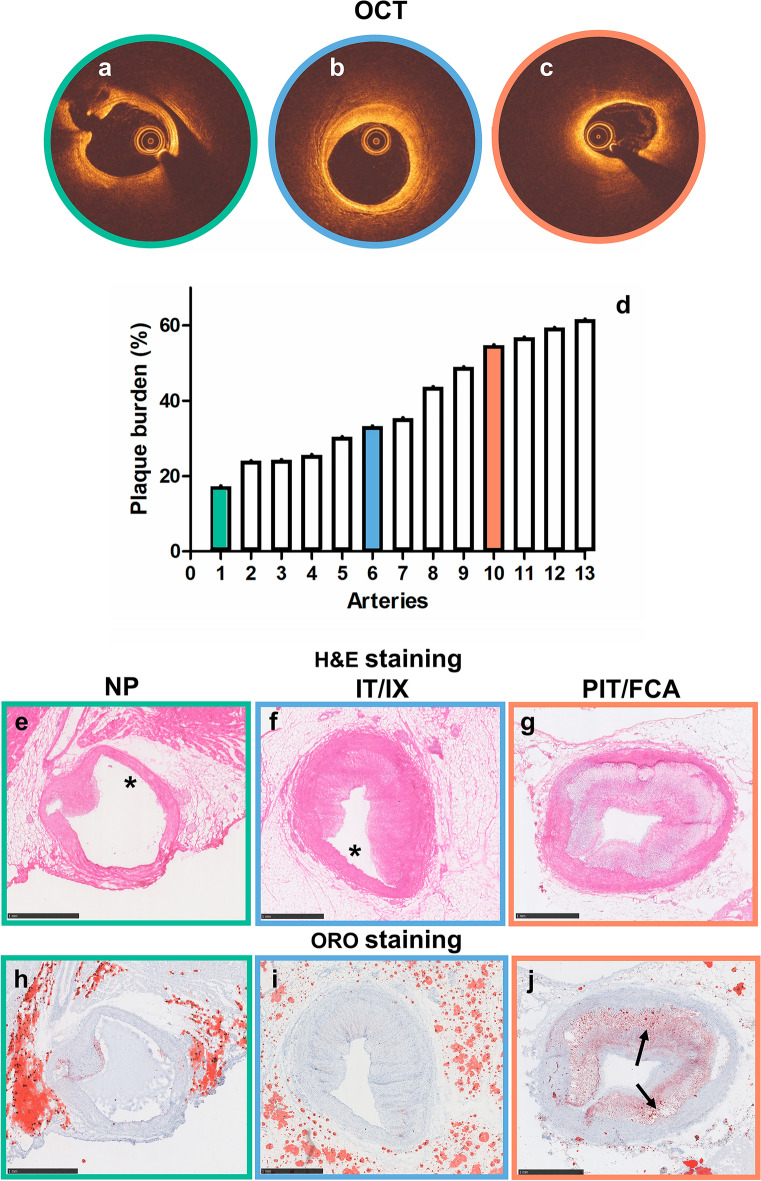
Histological verification of coronary atherosclerotic plaque type by OCT. Different types of plaques were selected by evaluation of OCT, which showed arteries without plaque (NP, **a** = **e** = **h**, green outline), with disease-free arcs (asterisk) and fibrous plaque (non-circumferential plaque) (IT/IX, **b** = **f** = **i**, blue outline) and with lipid-rich circumferential plaque (PIT/FCA, **c** = **g** = **j**, orange outline). The graph shows the distribution of plaque burden as evaluated by OCT (**d**). H&E (**e**–**g**) and ORO (**h**–**j**) staining of an artery without atherosclerotic plaque (**e**,**h**), with non-circumferential plaque (**f**,**i**) and with circumferential plaque (**g**,**j**). In ORO staining the lipids are stained in red (**j**); the asterisks indicate the plaque-free wall and the arrows indicate the lipid-rich regions. *NP* no plaque, *IT/IX* intimal thickening/intimal xanthoma, *PIT/FCA* pathological intimal thickening/fibrous cap atheroma.

In total, 13 coronary segments (3 mm) were analyzed by histology. Histology confirmed that the arteries presented a wide range of atherosclerotic plaques (Fig. 1a–d), ranging from segments without plaque (NP, n = 4, Fig. 1a,e,h), to fibrous plaques (IT/IX, n = 6, Fig. 1b,f,i), to lipid-rich circumferential plaques (PIT/FCA, n = 3, Fig. 1c,g,j).

### Pre-existing atherosclerotic plaque affects NI responses to DES

**Figure 2:**
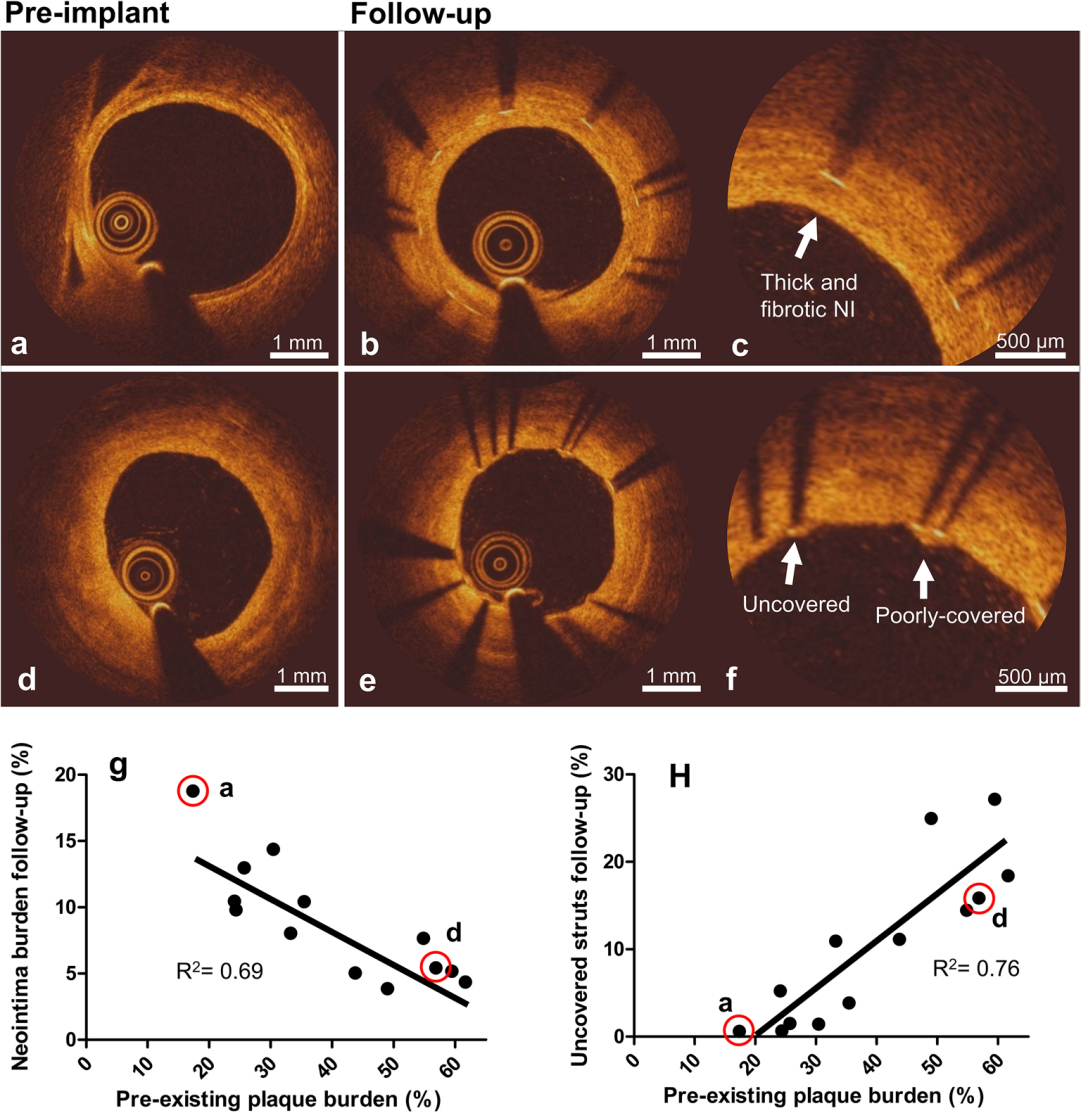
Relation between pre-existing plaque burden and strut coverage at follow-up as evaluated by OCT. Top: OCT frames showing an artery with low pre-implant plaque burden (**a**) and the same artery at follow-up (**b**), showing fibrotic neointima (NI) (close-up in **c**). Middle: OCT frames showing an artery with higher pre-implant plaque burden (**d**) and the same artery at follow-up (**e**), showing uncovered and poorly-covered struts (close-up in **f**). Bottom: Quantitative relations between pre-existing plaque burden and NI burden (**g**) and the percentage uncovered struts (**h**) at follow-up. The red rimmed dots designate the arteries shown in (**a**) and (**d**). While we present the data as a single data set based on non significant differences between the two stent types, full data including stent type are presented in Supplementary Fig. S3 for completeness.

The extent of pre-existing plaque affected the extent of strut coverage at follow-up (Fig. 2). Figure 2a shows a coronary artery with a very low pre-implant plaque burden, which resulted in high NI burden at follow-up (Fig. 2b and close-up in Fig. 2c), without the presence of uncovered struts. Figure 2d shows a coronary artery with a higher plaque burden, which resulted in minimal NI coverage and presence of uncovered struts at follow-up (Fig. 2e and close-up in Fig. 2f). Combining all data, a higher pre-implant plaque burden negatively correlated with NI burden (Fig. 2g) and positively correlated with the percentage uncovered struts (Fig. 2h), at follow-up.

**Figure 3:**
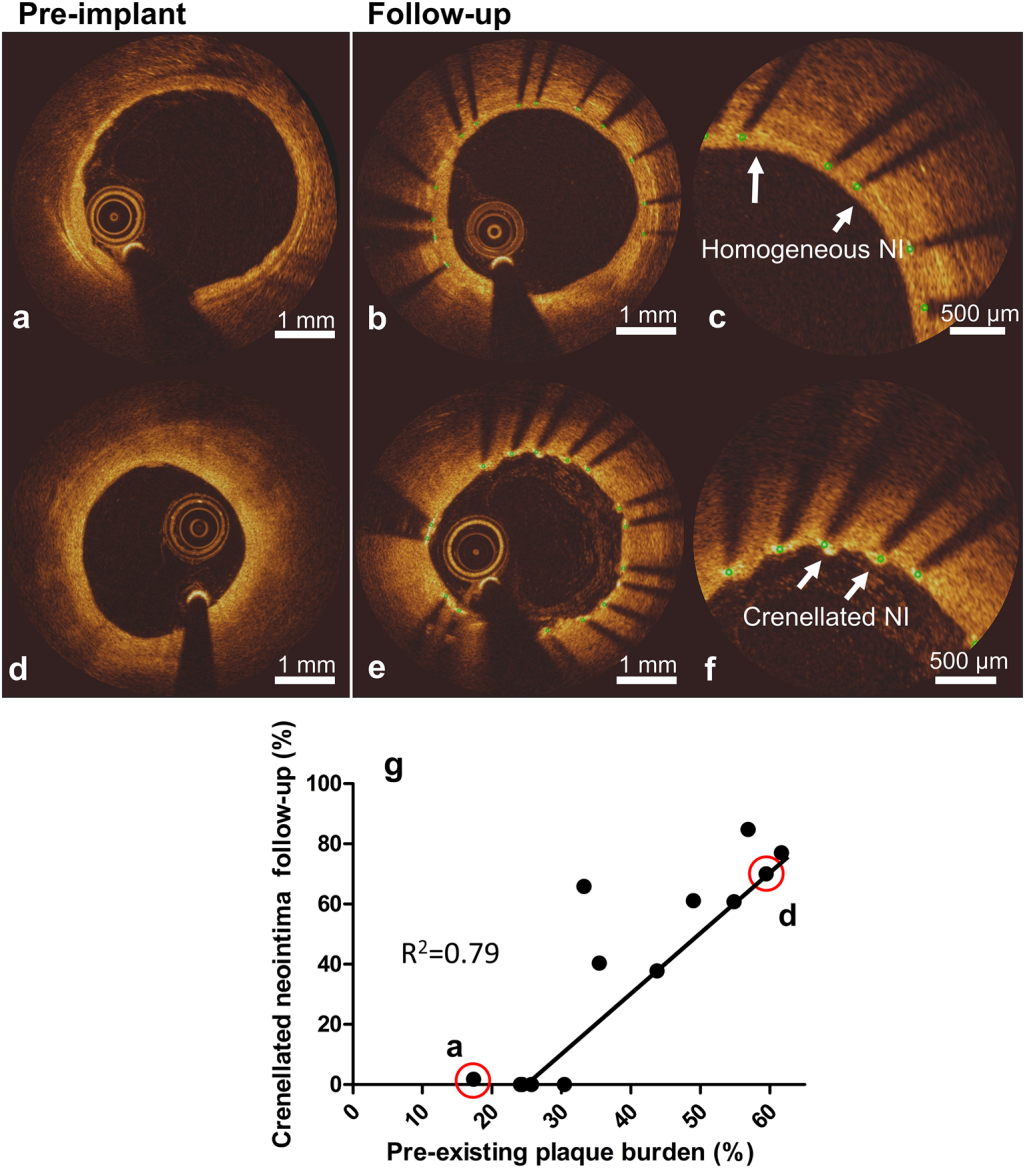
Relation between pre-existing plaque burden and pattern of strut coverage at follow-up as evaluated by OCT. Top: OCT frames showing an artery with low pre-implant plaque burden (**a**) and the same artery at follow-up (**b**), showing a homogeneous neointima (NI) (close-up in **c**). Middle: OCT frames showing an artery with higher pre-implant plaque burden (**d**) and the same artery at follow-up (**e**), showing crenellated NI (close-up in **f**). Bottom: quantitative relation between pre-existing plaque burden and percentage crenellated NI at follow-up (**g**). The red rimmed dots designate the arteries shown in (**a**) and (**d**). While we present the data as a single data set based on insignificant differences between the two stent types, full data including stent type are presented in Supplementary Fig. S3 for completeness.

Pre-implant plaque burden also influenced the type of strut coverage (Fig. 3). Coronary arteries with a very low pre-existing plaque burden (Fig. 3a) showed a homogeneous NI at follow-up (Fig. 3b and close-up in Fig. 3c). By contrast, coronary arteries presenting higher plaque burden (Fig. 3d) showed a crenellated NI at follow-up (Fig. 3e and close-up in f). More specifically, Fig. 3g shows a positive relation between the percentage of pre-existing plaque burden and the percentage of crenellated NI at follow-up. No signs of neoatherosclerosis were observed in all assessed frames.

**Figure 4:**
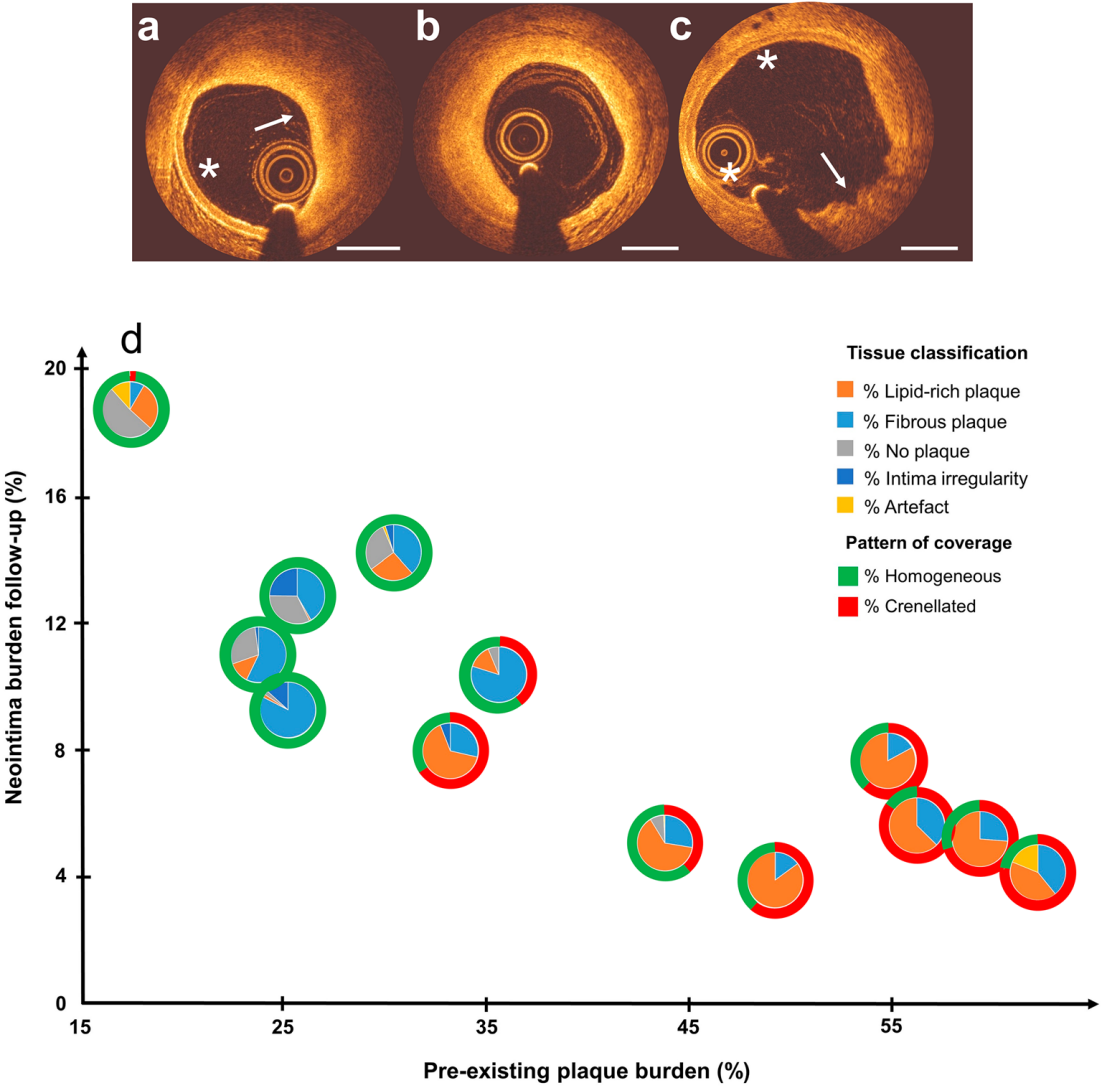
Contribution of tissue classification and pattern of coverage to the relation between pre-existing plaque burden and neointima (NI) burden at follow-up as evaluated by OCT. Top: plaque tissue was divided into lipid-rich (**a**, arrow), fibrotic (**b**), healthy (**a**, asterisk and **c**, asterisks) and intima presenting irregularity (**c**, arrow). Bottom: quantitative relation between pre-existing plaque burden and NI burden with the addition of tissue composition in the form of pie charts (**d**). Each pie chart indicates the percentage of tissue type for each stented artery (orange = lipid-rich, light blue = fibrous, grey = no plaque, blue = intima irregularity, yellow = artefact). The outer ring indicates the type of pattern of coverage (green = homogeneous NI, red = crenellated NI). The percentages of in-stent tissue composition pre-implant and the pattern of coverage at follow-up for each point shown in the figure are given in Supplementary Table S5. Scale bar: 1 mm.

Plaque composition was assessed pre-implantation. The upper panel in Fig. 4 reports examples of lipid-rich plaque (Fig. 4a), fibrous plaque (Fig. 4b), healthy vessel and intima irregularity (Fig. 4c). The graph (Fig. 4d) shows the relation between pre-implant plaque burden and follow-up NI as before, but now with information on plaque composition added. The internal pie chart of each point shows the percentage of tissue composition averaged per stent, while the external ring indicates the pattern of strut coverage at follow-up. Simple linear regression analyses of this data shows that the percentage of lipid-rich plaque was a predictor for NI burden, percentage of uncovered struts and crenellated pattern of coverage, while the percentage of fibrous plaque was not (Supplementary Table S5).

## Discussion

### Pre-existing atherosclerotic plaque and NI response to DES

Following DES implantation, human coronary arteries often present uncovered or poorly-covered struts at follow-up^21,22^, while commonly used healthy coronary swine models usually do not^10,11,23^. The reason for this difference in behavior is not well understood, but we hypothesized that swine models with significant pre-existing plaque would show responses following DES implantation more similar to patients, especially with regard to the presence of poor strut coverage. To test this hypothesis, we specifically chose to use an adult FH swine model, which reported the presence of a wide range in plaque burden, from healthy segments (NP), non-atherosclerotic intimal lesions (IT/IX), to progressive atherosclerotic lesions (PIT/FCA)^13,20,24^.

So far, few studies addressed the NI response following DES implantation in FH or other diseased swine models as studied by OCT^12,25–27^. These studies used relatively young animals (~ 8 months) and show relatively low plaque burden resulting in thick homogeneous NI at follow-up (see Supplementary Table S7). In contrast, we used adult animals (~ 42 months) with a wider range in plaque severity, including advanced plaques. For lower plaque burden, our observations for NI response are in line with the above-mentioned studies. For higher plaque burden, we reveal human-like responses, with minimal NI thickening and uncovered struts. While this confirms our hypothesis, our work also reveals two important aspects of the response to DES: (1) NI thickness is negatively associated with plaque burden (2) Plaque composition and in particular the presence of a lipid-rich plaque leads to more poorly-covered and uncovered struts.

### Clinical relevance

It was previously shown that the development of plaque in this adult FH disease model was human-like, but the response to DES implantation was not yet studied. This study demonstrates that the NI response to DES implantation is also human-like, further underlining the clinical importance of this adult FH swine model.

More specifically, we found that NI thicknesses at 28 days following DES implantation range between 72 and 204 µm, with lower values in the presence of larger underlying plaque burden. These values are comparable to findings from clinical studies, with the NI thicknesses ranging between 40 and 164 µm at follow-up (2 weeks to 9 months)^9,21,28–31^. In addition, uncovered struts and crenellated NI, considered two important parameters of impaired healing following DES, were also present. In patients, the percentage of uncovered struts at follow-up varies between 2 and 28%^29,31^. In our study, we found similar percentages ranging between 0.6 and 27%. These findings show that the presence of biologically relevant plaques in a swine model is crucial to better reproduce the clinical situation.

With respect to plaque composition and how it influences NI responses, only two OCT studies reported data following DES in patients. They report that human coronary arteries show thicker NI in the presence of lipid-rich plaques, as compared to struts laying on fibrous plaques^32,33^. Our results in Fig. 4 seem to show the opposite. NI burden was lower when the stent was implanted in a lipid-rich segment, while NI burden was higher when the stent was implanted in a healthy segment or presenting a fibrous plaque. We attribute this apparent difference to the difference in range of plaque burden over which the NI response was observed, and to differences in plaque characteristics between our study and previously published work. The above-mentioned studies analyzed NI thickness in patients with advanced plaques, whereas our study focused on mildly to moderately advanced plaques in a specific swine model.

Finally, we note that this study revealed that larger plaque burden upon DES implantation results in thinner NI and larger percentages of uncovered struts. In this light, the current model, presenting relevant pre-existing plaque burden, underlines why uncovered struts are not usually seen in healthy or minimally diseased pre-clinical coronary models.

### Study limitations

First, the observed plaque burden did not reach the amount reported in patients presenting for percutaneous coronary intervention (PCI) (around 90%). In our animals, plaque burden reached up to 60%. However, this lower plaque burden did allow us to visualize the EEL and determine plaque burden by OCT. Out of 1074 in-stent frames analyzed, EEL was not completely visible in only 73 frames. Second, our follow-up period was restricted to 28 days after DES implantation. Thus, we cannot draw conclusions on the vascular response beyond this time point. However, the 28-day time point is an accepted standard in porcine models and allows comparison of our results with previous published work. Moreover, at 28 days, a significant amount of drug has been released from the stents used in our study, approximately 50% for both cases. This allowed us to study the NI response in the presence of a stable drug release. While the addition of multiple time-points would provide a more comprehensive understanding of NI responses, this would require significant financial resources. Finally, even though the here presented swine model represent a unique model to study responses to DES in advanced plaques, the practicality of this model is limited due to time and costs associated.

## Conclusions

In the studied FH swine model, NI responses present the whole spectrum that is seen in patients: uncovered struts, poorly-covered struts, and fibrotic NI. Interestingly, percentage of pre-existing plaque burden is negatively associated with NI burden. Particularly, the presence of a lipid-rich plaque results in more poorly-covered and uncovered struts, which underscores the importance of advanced disease when performing safety and efficacy testing of DES.

## Supplementary Information

Supplementary Information.

## Data Availability

The datasets generated during and/or analysed during the current study are available from the corresponding author on reasonable request.
