# Supplementary material for: Plaque burden is associated with minimal intimal coverage following drug-eluting stent implantation in an adult familial hypercholesterolemia swine model
Source: Sci Rep. 2023 Jul 1;13:10683. doi: 10.1038/s41598-023-37690-0 (PMC10314904; doi:10.1038/s41598-023-37690-0)
Supplement: Supplementary file 1 — Supplementary Information. [file 41598_2023_37690_MOESM1_ESM.pdf]

SUPPLEMENTARY MATERIALS

Plaque burden is associated with minimal intimal coverage following drug-eluting stent implantation in an adult familial hypercholesterolemia swine model

Francesca Razzi, M.Sc.,[a] Jouke Dijkstra, Ph.D.,[b] Ayla Hoogendoorn, Ph.D.,[a] Karen Witberg, R.N.,[a] Jurgen Ligthart, B.Sc.,[a] Dirk J. Duncker, M.D., Ph.D.,[a] Jan van Esch, Ph.D.[c], Jolanda J. Wentzel, Ph.D.,[a] Volkert van Steijn, Ph.D.,[c] Gijs van Soest, Ph.D.,[a] ,Evelyn Regar, M.D., Ph.D.,[d*], Heleen M.M. van Beusekom, Ph.D.[a*]

[a]Dept. Cardiology, Erasmus MC, Rotterdam, The Netherlands

[b]Leiden University Medical Center, Leiden, The Netherlands

[c]Delft University of Technology, Delft, The Netherlands

[d]University Hospital Ludwig-Maximilians, Munich, Germany.

*, These authors contributed equally

Supplementary Methods

Tissue classification: Lipid-rich plaque was defined as tissue showing an inhomogeneous fading signal combined with an absent 3-layered structure. Fibrous plaque was defined as tissue with high backscattering and a relatively homogeneous signal, at the luminal side of the internal elastic lamina (IEL). Healthy vessel was defined as a vessel with a normal 3-layered structure, with a maximal intimal thickness of 30 μm. Intima irregularity was defined as a region of the vessel wall where the intima was irregular or dissected either due to the development of the disease or due to instrumentation. Other was defined as an arc in which the tissue composition was not identifiable (i.e. presence of blood, thrombus, guidewire or optical artefacts).

Supplementary Figures

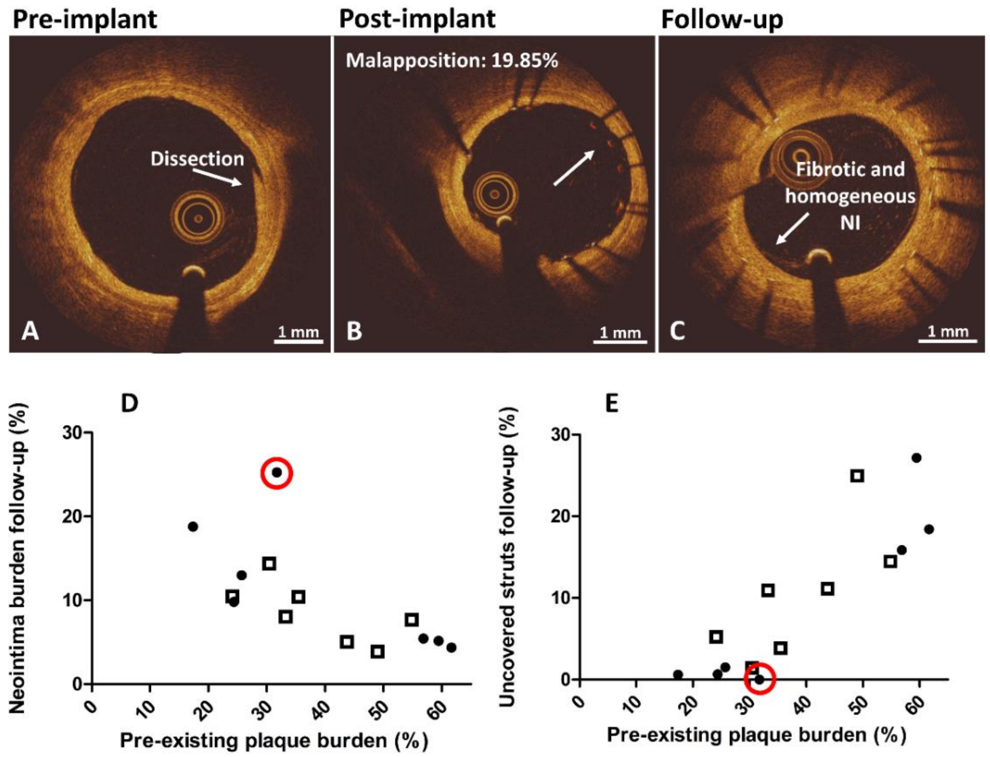

Figure S1: optical coherence tomography (OCT) analysis of the stent excluded from the analysis. Top: OCT cross sections showing the artery with a long dissection pre-implant (A, arrow), resulting in excessive malapposition post-implant (B, arrow) and a fibrotic and homogeneous neointima (NI) at follow-up (C, arrow). Bottom: Quantitative relation between pre-existing plaque burden and NI burden at follow-up (D). Quantitative relation between pre-existing plaque burden and uncovered struts at follow-up (E). Circle = ORSIRO stent, square = MISTENT stent. The red circles designate the artery excluded from the analysis.

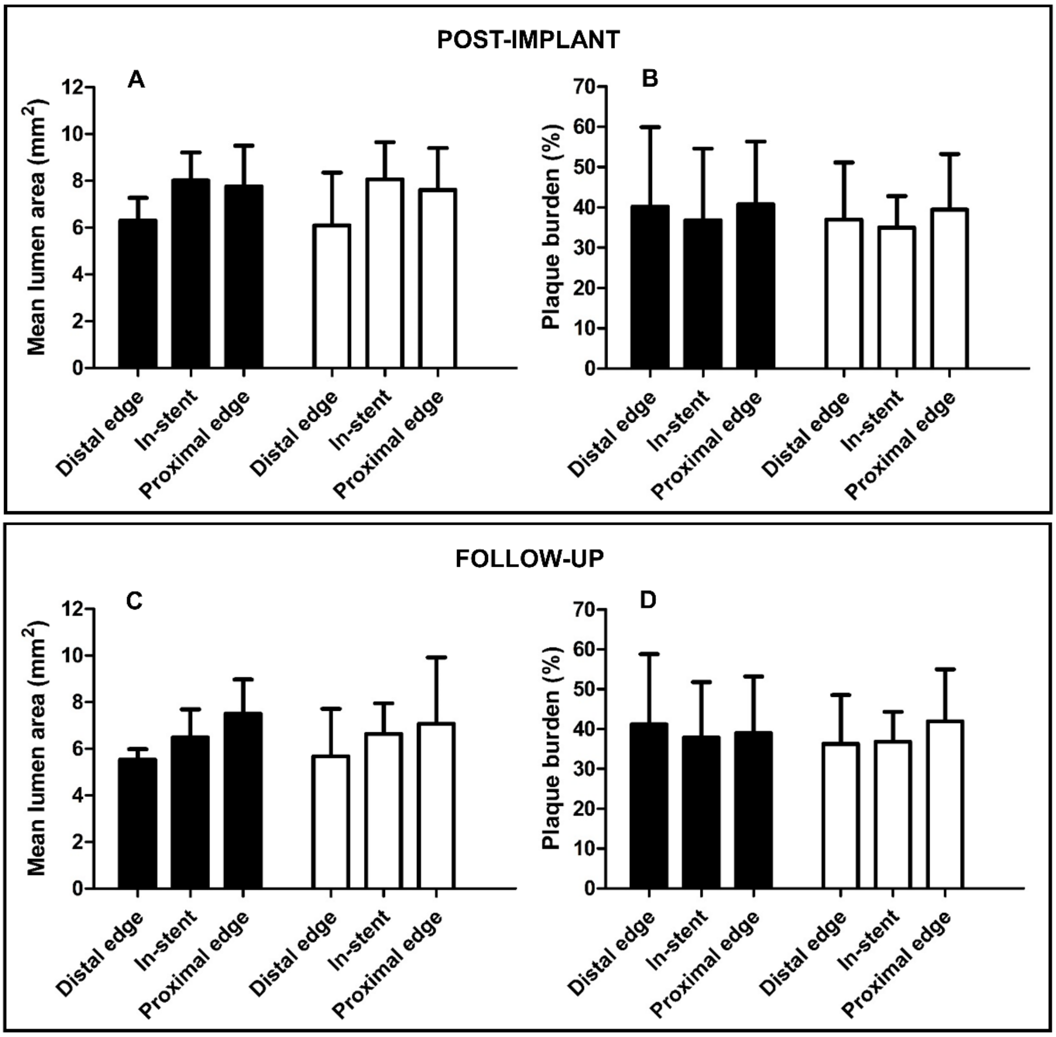

Figure S2: Mean lumen areas and percentage plaque burden in the stented segments and in the distal/proximal reference segments evaluated by optical coherence tomography (OCT). Top: post-implant mean lumen areas (A) and plaque burden (B) for ORSIRO (black) and MISTENT (white) groups. Bottom: follow-up mean lumen areas (C) and plaque burden (D) for ORSIRO (black) and MISTENT (white) groups.

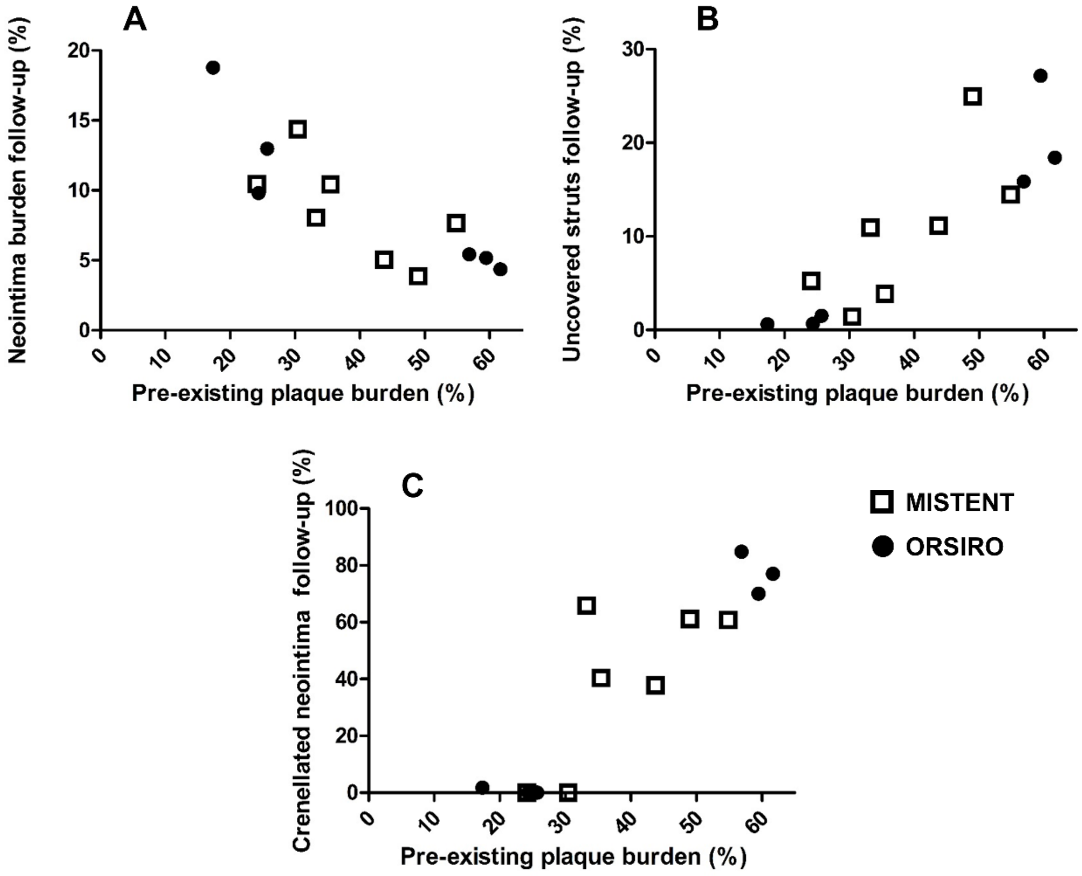

Figure S3: Relation between pre-existing plaque burden and neointima (NI) burden (A), percentage of uncovered struts (B) and crenellated pattern of strut coverage (C) at follow-up as evaluated by optical coherence tomography (OCT). Circle = ORSIRO stent, square = MISTENT stent.

Supplemental Tables

	Weight (kg)	Cholesterol (mmol/L)
Animal 1	59	12.7
Animal 2	69	5.5
Animal 3	69	16.9
Animal 4	85	15.3
Animal 5	76	17.7
Animal 6	76	16.2

Table S1: FH swine characteristics at baseline

		**ORSIRO**	**MISTENT**	**p-value**
**Pre-implant**	**n**	6	7	
	**Min lumen area (mm^2)**	6.13 ± 1.55	5.61 ± 1.51	0.558
	**Mean lumen area (mm^2)**	6.86 ± 1.36	7.12 ± 1.14	0.707
	**% plaque burden**	41 (17-62)	35 (24-55)	0.808
**Post-implant**	**n**	6	7	
	**Min lumen area (mm^2)**	7.86 ± 1.17	7.66 ± 1.39	0.789
	**% acute gain**	17 (11-47)	32 (10-42)	0.526
	**Mean lumen area (mm^2)**	8.01 ± 1.20	8.06 ± 1.59	0.944
	**Mean stent area (mm^2)**	8.03 ± 1.25	8.15 ± 1.62	0.883
	**Min stent area (mm^2)**	6.79 ± 1.44	6.72 ± 1.34	0.927
	**% plaque burden**	36 (19-55)	35 ± 8	0.823
**28 days follow-up**	**n**	6	7	
	**Min lumen area (mm^2)**	6.66 ± 1.32	6.33 ± 1.23	0.657
	**Mean lumen area (mm^2)**	6.49 ± 1.21	6.63 ± 1.32	0.838
	**Mean stent area (mm^2)**	7.17 ± 1.33	7.24 ± 1.28	0.927
	**Min stent area (mm^2)**	6.55 ± 1.25	6.73 ± 1.56	0.824
	**Late loss (%)**	11 (4-43)	6 (-23-39)*	0.405
	**% plaque burden**	36 (22-54)	37 ± 8	0.862
	**% NI burden**	8 (4-19)	8 (4-14)	0.741

Table S2: Optical coherence tomography (OCT) in-stent analysis of ORSIRO and MISTENT separately, pre-implant, post-implant and at 28 days follow-up. Results are reported as mean ±standard deviation or median (min-max). There were no differences between the two stent types for all the reported parameters. * negative values were present as some arteries showed a larger lumen area at follow-up as compared to post-implant. NI = neointima.

		ORSIRO	MISTENT	p-value
**Post-implant**	**n struts**	5504	11793	
	**% malapposition**	4.6 (0.2-17.7)	0.5 (0.1-3.9)	0.095
**Follow-up**	**n struts**	5214	8746	
	**NI thickness (µm)**	114 ±51	104 ±25	0.659
	**% uncovered struts**	9 (1-27)	11 (1-25)	0.940

Table S3: Strut-by-strut optical coherence tomography (OCT) in-stent analysis of ORSIRO and MISTENT separately, pre-implant, post-implant and at 28 days follow-up. Results are reported as average ±standard deviation or median (min-max). There were no differences between the two stent types for all the reported parameters. NI = neointima.

**Post implant**					
		All	ORSIRO	MISTENT	p-value
Distal edge	n	13	6	7	
	Mean lumen area (mm^2)	6.19 ± 1.72	6.30 ± 0.97	6.09 ± 2.26	0.839
	% plaque burden	43 (16-61)	41 (16-61)	43 (19-56)	0.740
Proximal edge	n	13	6	7	
	Mean lumen area (mm^2)	7.69 ± 1.69	7.77 ± 1.73	7.62 ± 1.79	0.882
	% plaque burden	40 ± 14	41 ± 16	39 ± 14	0.872
**28 days follow-up**					
		All	ORSIRO	MISTENT	p-value
Distal edge	n	13	6	7	
	Mean lumen area (mm^2)	5.61 ± 1.46	5.53 ± 0.45	5.68 ± 2.03	0.860
	% plaque burden	36 (20-58)	43 (20-58)	36 (24-54)	0.566
Proximal edge	n		6	7	
	Mean lumen area (mm^2)	7.27 ± 2.24	7.50 ± 1.47	7.07 ± 2.85	0.742
	% plaque burden	41 ± 13	39 ± 14	42 ± 13	0.703

Table S4: OCT analysis of the segments 5 mm distal and proximal outside the stent edges for the ORSIRO and MISTENT groups. Results are reported as average ±standard deviation or median (min-max).

**Simple linear regression**			
**Dependent variable**	**Independent variable**	**P value ANOVA**	**R^2**
NI burden	Plaque burden	**0.0004**	0.690
	% lipid-rich plaque	**0.015**	0.430
	% fibrous plaque	0.813	0.005
% uncovered struts	Plaque burden	**0.0001**	0.760
	% lipid-rich plaque	**0.0004**	0.690
	% fibrous plaque	0.111	0.210
Crenellated pattern of coverage	Plaque burden	**0.00004**	0.79
	% lipid-rich plaque	**0.001**	0.62
	% fibrous plaque	0.272	0.12
Plaque burden	Blood cholesterol	0.727	0.011
	Weight	**0.028**	0.341

Table S5: Simple linear regression of the variables analyzed. NI = neointima

**Pre-implant**						**Follow-up**		
**%PB**	**%LR**	**%F**	**%NP**	**%II**	**%O**	**%NI**	**%H-NI**	**%C-NI**
17	28	8	52	0	12	19	98	2
24	12	57	28	2	0	10	100	0
24	2	82	2	13	0	10	100	0
26	1	42	33	25	0	13	100	0
30	27	38	29	5	2	14	100	0
33	66	29	0	6	0	8	34	66
35	14	80	6	0	0	10	60	40
44	63	27	8	0	1	5	62	38
49	85	15	0	0	0	4	39	61
55	83	17	0	0	0	8	39	61
57	63	37	0	0	0	5	15	85
59	74	26	0	0	0	5	30	70
62	42	39	0	0	19	4	23	77

Table S6: OCT analysis of in-stent percentage of tissue composition for each stent per row. PB = plaque burden; LR = lipid-rich; F = fibrous; NP = no plaque; II = intima irregularity; O = others; NI = neointima; H-NI = homogeneous neointima; C-NI = crenellated neointima.

Animals (age, months)	NI thickness (µm)	Stent	Analysis	Time point	Reference
FH swine (~8)	400 ± 100	EES	OCT	28 days	[1]
FH swine (~8)	300 ± 20	EES	OCT	30 days	[2]
FH swine (~8)	210 (150 – 310)	PES	OCT	30 days	[3]
Yorkshire DM/HC (~9)	230 ± 50	PES	Histology	30 days	[4]
FH swine (43)	110 (72-204)	SES	OCT	28 days	Current model

Table S7: Follow-up neointima (NI) thickness after drug-eluting stent (DES) implantation in diseased swine models, approximately 8 months old. Values of neointima (NI) are reported as average ± standard deviation or median (min-max). OCT = optical coherence tomography; FH = familial hypercholesterolemia; DM = diabetes mellitus; HC = hypercholesterolemia; EES = Everolimus-eluting stent; PES = Paclitaxel-eluting stent; SES = Sirolimus-eluting stent.

Supplementary references

1. Tellez, A. *et al.* Experimental evaluation of efficacy and healing response of everolimus-eluting stents in the familial hypercholesterolemic swine model: A comparative study of bioabsorbable versus durable polymer stent platforms. *Coron. Artery Dis.* **25**, 198–207 (2014).
2. Tellez, A. *et al.* Peri-strut low-intensity areas in optical coherence tomography correlate with peri-strut inflammation and neointimal proliferation: An in-vivo correlation study in the familial hypercholesterolemic coronary swine model of in-stent restenosis. *Coron. Artery Dis.* **25**, 595–601 (2014).
3. Gasior, P. *et al.* Impact of Fluoropolymer-Based Paclitaxel Delivery on Neointimal Proliferation and Vascular Healing. *Circ. Cardiovasc. Interv.* **10**, (2017).
4. Llano, R. *et al.* Vascular responses to drug-eluting and bare metal stents in diabetic/hypercholesterolemic and nonatherosclerotic porcine coronary arteries. *Circ. Cardiovasc. Interv.* **4**, 438–446 (2011).
